# Comparing Email, SMS, and Concurrent Mixed Modes Approaches to Capture Quality of Recovery in the Perioperative Period: Retrospective Longitudinal Cohort Study

**DOI:** 10.2196/25209

**Published:** 2021-11-04

**Authors:** Jamie L Romeiser, James Cavalcante, Deborah C Richman, Sunitha M Singh, Xiaohui Liang, Allison Pei, Samanvaya Sharma, Zoe Lazarus, Tong J Gan, Elliott Bennett-Guerrero

**Affiliations:** 1 Department of Anesthesiology Stony Brook University Medical Center Stony Brook, NY United States

**Keywords:** concurrent mixed modes, recovery after surgery, text messages, SMS, email, perioperative recovery, mobile phone

## Abstract

**Background:**

As patients are discharged from the hospital more quickly, the ability to monitor patient recovery between hospital discharge and the first follow-up clinic visit is becoming increasingly important. Despite substantial increase in both internet use and smartphone ownership over the past 5 years, clinicians have been slow to embrace the use of these devices to capture patient recovery information in the period between hospital discharge and the first clinical follow-up appointment.

**Objective:**

This study aims to investigate the generalizability of using a web-based platform to capture patient recovery in a broad surgical patient population and compare response rates for 3 different web-based strategies for delivering recovery surveys over the perioperative period: email, SMS text messaging, and a concurrent mixed approach of using both email and SMS text messaging.

**Methods:**

Patients undergoing surgeries managed with an enhanced recovery after surgery pathway were asked to participate in a web-based quality assurance monitoring program at the time of their preoperative surgery appointment. Different follow-up methods were implemented over 3 sequential phases. Patients received Health Insurance Portability and Accountability Act–compliant web-based survey links via email (phase 1), SMS text messaging (phase 2), or concurrently using both email and SMS text messaging (phase 3) using REDCap and Twilio software. Recovery assessments using the established Quality of Recovery-9 instrument were performed 4 days before surgery and at 7 and 30 days postoperatively. Generalizability of the web-based system was examined by comparing characteristics of those who participated versus those who did not. Differences in response rates by the web-based collection method were analyzed using adjusted models.

**Results:**

A total of 615 patients were asked to participate, with 526 (85.5%) opting for the follow-up program. Those who opted in were younger, slightly healthier, and more likely to be in a partnership. The concurrent mixed modes method was the most successful for obtaining responses at each time point compared with text or email alone (pre: 119/160, 74.4% vs 116/173, 67.1% vs 56/130, 43.1%, *P*<.001; 7 days: 115/172, 66.9% vs 82/164, 50.0% vs 59/126, 46.8%, *P*=.001; 30 days: 152/234, 65.0% vs 52/105, 49.5% vs 53/123, 43.1%, *P*=.001, respectively). In the adjusted model, the concurrent mixed modes method significantly predicted response compared with using email alone (odds ratio 3.4; *P*<.001) and SMS text messaging alone (odds ratio 1.9; *P*<.001). Additional significant predictors of response were race, partnership, and time.

**Conclusions:**

For internet users and smartphone owners, electronic capture of recovery surveys appear to be possible through this mechanism. Discrepancies in both inclusion and response rates still exist among certain subgroups of patients, but the concurrent approach of using both email and text messages was the most effective approach to reach the largest number of patients across all subgroups.

## Introduction

### Background

Patients are discharged from the hospital more quickly with the help of minimally invasive procedures, safer and shorter acting drugs, enhanced recovery programs, and advances in patient safety and monitoring [1,2]. There are several benefits of a reduced length of hospital stay, including reductions in nosocomial infections [3], opioid use, and medical expenditures [2]. However, this also means that a larger proportion of the patient’s recovery process is spent at home. What happens in the short-term recovery process, that is, the time between the time of discharge and the first clinical follow-up appointment, can be crucial for setting the trajectory of a patient’s long-term recovery after surgery [4].

Patient-reported outcomes (PRO) are an important component of clinical outcomes [5-7]. The collection of PROs, especially once a patient has been discharged, is challenging and is thus underutilized in clinical practice [8]. Finding a practical and affordable way to contact patients outside of a clinical setting is challenging. Sending postal surveys leads to a large delay in the return of information, and repeated telephone calls require a significant amount of time and resources. A more practical solution would be to use a web-based approach. It is estimated that 90% of adults in the United States use the internet on a regular basis, and 81% own a smartphone [9].

The use of this technology is at an all-time high in the United States, but it is not often leveraged to implement short-term postdischarge recovery monitoring programs. Patient demographics, such as age [10], can be a barrier for inclusion in recovery monitoring programs that use SMS-text. High response rates have been demonstrated for these types of SMS programs [11-13], but many of these studies did not report the number of patients or demographics of patients who were excluded from the intervention. Other studies that focus on long-term recovery surveys (ie, 3 months-2 years) have found some success with using email methods to deliver these surveys [14,15]. However, these studies used secondary methods, such as telephone reminders and postal questionnaires, to obtain responses. Patients cited lack of email, lack of internet access, infrequent email use, or browser incompatibility as reasons for not completing web-based questionnaires [14], and response rates have been shown to differ by race, household income, and procedural characteristics [15].

### Objectives

The ability to maximize participation and engagement to produce generalizable and unbiased information for quality-of-care improvement is essential. One approach that is rarely considered is to send PRO recovery surveys through a concurrent mixed-mode approach, which uses a combination of simultaneous email and text messages. Thorough examination of the generalizability and feasibility of such a system in the short-term perioperative period has not been explored. To our knowledge, response rates using email alone, text alone, and this combined approach have not been directly compared in this setting. Therefore, the primary aim of this study is to evaluate and compare the feasibility of collecting short-term perioperative PROs within an enhanced recovery after surgery (ERAS) population using 3 different web-based and mobile phone collection mechanisms—email alone, text alone, and a concurrent mixed modes (CMM) approach.

## Methods

### Study Design and Participants

This project follows the Standards for Quality Improvement Reporting Excellence (SQUIRE) guidelines. Although this project was authorized by the institution’s Division of Medical and Regulatory Affairs Office as part of its Surgical Quality Improvement Program, following deidentification and extraction of all Surgical Quality Improvement Program patient records, data analyses were performed as part of the Surgical Quality Data Users Group. The Surgical Quality Improvement Program/Surgical Quality Data Users Group protocols were approved by our institution’s review board (Committee on Research Involving Human Subjects, CORIHS #170753-13).

The longitudinal cohort consisted of surgical patients managed by an ERAS pathway. This quality assurance (QA) program includes patients who are undergoing colorectal, minimally invasive gynecology, orthopedic, plastic, lumbar spine, urology, surgical oncology, and urogynecology-related surgeries. In 2018, the program implemented a web-based element in which patients could opt-in and complete follow-up questionnaires regarding their recovery after surgery. All patients who were treated using the ERAS pathway were eligible to participate in the follow-up portion of the QA program. Opt-in forms were completed by the patients during their preoperative services visit.

### Survey Instrument, Software, and Distribution

We used the Quality of Recovery-9 (QOR-9) survey, which was developed specifically to quantify overall recovery status after surgery and anesthesia [16,17], and has been validated in a diverse set of surgical patients both preoperatively and postoperatively at time points ranging from 1 day to 6 weeks [17]. REDCap (Vanderbilt University) software with Twilio (Twilio Inc) application integration was used for the survey distribution. Twilio is a separate third-party app that can send text messages with survey links. We used the social exchange theory to guide several survey design choices, such as including a welcome message with information about patient privacy, adding an institutional logo, using a QA-specific email address, and ensuring that the survey was brief. Surveys were compatible with all types of browsers and devices, and fonts and colors were accessible to individuals who may have had a vision impairment.

Automated surveys were programmed to be sent to patients based on their procedure date. Surveys were sent through Health Insurance Portability and Accountability Act–compliant survey links, even though surveys did not contain any protected health information or personally identifiable information. Three time points were chosen to administer the QOR-9 survey: 4 days before the patient’s planned procedure date and at 7 and 30 days after the planned procedure date. Surveys were delivered by a different method of contact across 3 sequential phases over a 6-month time span. Patients who underwent surgery in the first phase were preassigned to receive the surveys by email link alone; patients who had surgery in the second phase were preassigned to receive the surveys by a text link alone; and patients who had surgery in the third phase were preassigned to receive the surveys through a CMM approach, and they received both an email link and a text link at the same time. If participants only listed one mode of contact (ie, only email or only smartphone number), the survey was sent to that method of contact regardless of the preassignment. This was done to prevent any exclusion from the QA program. Analyses were run as *per protocol* analyses, with the actual method of contact as the primary predictor in the response models.

All surveys were sent at 9:15 AM local time. One automated reminder per survey was programmed into the REDCap software and sent to the patients who had not responded to the original survey within 24 hours. At the preoperative and 7-day postoperative time points, a follow-up phone call was also made if patients did not respond within 24 hours to the automated reminder. All survey links expired after 3 days.

### Study Variables

#### Method of Contact

The primary predictor of response was the method of contact. The method of contact used to send survey links to participants was either through email alone, SMS text messaging alone, or through both an email and SMS simultaneously (CMM).

#### Outcomes

The primary outcome was response to the survey questionnaires. Because the primary purpose of this study was to examine the feasibility of a web-based approach to obtain recovery information, responses were only considered positive if they were obtained by the web-based system. Responses obtained through telephone calls were characterized as a nonresponse. Response variables were created separately for each time point (preoperatively, 7, and 30 days). Generalizability (ie, inclusion) was a secondary outcome and was defined as opting into the follow-up program versus opting out.

#### Demographic Characteristics

Individual-level sociodemographic characteristics, including age, race, ethnicity, and partnership (marital status), were extracted from the hospital’s electronic medical record for all ERAS QA program participants. Age was examined continuously but was also dichotomized into <75 and ≥75 years old. This dichotomy was chosen as previous literature describes a noted decline in the use of web technology in individuals 75 years of age and above [18]. Partnership was defined as either being currently married or in a partnership. The primary language was categorized as English and non-English speaking.

#### Clinical Characteristics

Each element of the Charlson comorbidity index (CCI) was collected from the electronic medical record according to the definitions outlined in the index instructions [19]. Age was not included in the index calculation to avoid multicollinearity. A CCI score higher than 3 was rare; therefore, for ease of interpretation and model stability, the index was categorized into 4 groups: 0, 1, 2, and 3+. Additional key comorbidities, such as depression and anxiety (defined as an active diagnosis or depression medication within 3 months of surgery) and chronic pain (defined as an active diagnosis or opioid prescription within 3 months of surgery), were extracted from the preoperative service visit records in the electronic medical record.

#### Population-Level Characteristics

Zip code level factors, including inflation-adjusted median income and proportion of residents with greater than a high school education, were obtained from the United States Census Bureau’s 2017 American Community Survey 5-year estimates.

### Statistical Analysis

Demographics and clinical comorbidities were compared between those who opted in and those who opted out of the follow-up program using both univariate and multivariable logistic regression models. Similar analyses were performed between those who responded and those who did not at each time point, with the method of contact as the primary predictor of interest. All time point data were then combined for a final multivariable model using generalized estimating equations with an independent correlation structure to adjust for repeated measures. Multicollinearity was present for education and median income zip code level factors in all analyses; therefore, only median income was used in the multiple regression models. Sensitivity analyses were performed to address any effects that coverage error may have on the nonresponse analyses by using inverse probability weighting. Inverse probability weighting is a technique used to make the response sample (ie, those who opted in) more reflective of the original ERAS population. Weights were calculated on the basis of the probability of inclusion from the adjusted logistic regression model in the generalizability analysis and applied to the *opt-in* population for a weighted generalized estimating equations sensitivity analysis [20-22].

A common concern in survey research is nonresponse bias, as it relates to missing outcomes. One strategy used to overcome this issue is to develop a proxy group for nonresponders, which can be based on the continuum of resistance theory [23,24]. This theory states that late responders have similar characteristics and outcomes as nonresponders [25]. Therefore, we created a variable that indicated *early* responders (ie, response on the first request), *delayed* responders (ie, response to the 24-hour reminder), and *late* responders (ie, those who responded to the telephone call) to examine nonresponse bias. QOR-9 scores were first tested for normality. Data were not normally distributed (Shapiro–Wilk test *P*<.05); therefore, QOR-9 scores between the 3 response groups were compared using a Kruskal–Wallis test. Tests were performed separately for the preoperative and 7-day postoperative time points.

Three separate Blinder-Oaxaca decomposition analyses were performed to further characterize any differences in the composition of the *email alone* group compared with the *SMS alone* group, CMM compared with the SMS alone group, and CMM compared with the email alone group. Blinder-Oaxaca decomposition analyses were performed using STATA’s (version 15, Stata Corp) mvdcmp command with logistic regression. All other analyses were performed using SAS version 9.4 (SAS Institute Inc) at 95% CI.

## Results

### Description of Patient Characteristics

**Figure 1:**
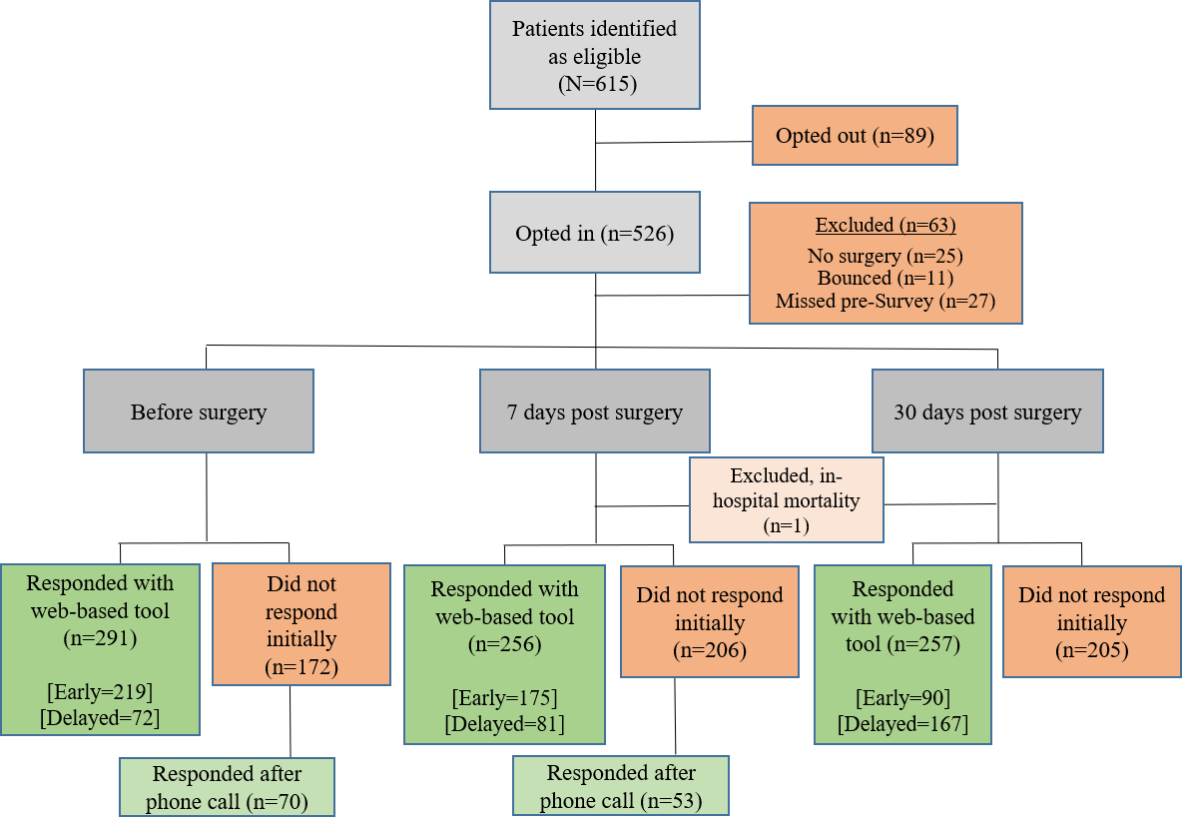
Flow diagram of enhanced recovery after surgery participants. Patients who opted in (n=526) and those who opted out (n=89) were compared in the generalizability analyses. Those who opted in and were not excluded for other reasons were examined in the response analyses (n=463). Those who responded after the phone call were not considered responders in the primary response analysis because they did not use the web-based tool to respond; instead, these individuals were included as late responders in the Quality of Recovery score analyses that examined nonresponse bias within outcomes.

A flow diagram of the participants is shown in Figure 1. A total of 615 patients were eligible: 526 (85.5%) opted-in to the additional follow-up program and 89 (14.5%) either declined or did not have an email address or smartphone. An additional 63 participants were excluded from the response analyses for various reasons. The characteristics of the patients with ERAS are described in Table 1*.*

**Table 1:** Demographic and clinical characteristics of the enhanced recovery after surgery population.

Characteristics					All (N=615)			Opted-in (n=526, 85.5)			Opted-out (n=89, 14.5)			*P* value
**Demographics**														
	Sex (female), n (%)			404 (65.7)			345 (65.6)			59 (66.3)			.90^a^	
	Age (years), mean (SD)			62.6 (12.0)			61.64 (11.5)			68.21 (13.5)			<.001^b^	
	Age (≥75 years), n (%)			95 (15.5)			61 (11.6)			34 (38.2)			<.001^a^	
	Race/ethnicity (non-Hispanic White), n (%)			533 (86.7)			457 (86.9)			76 (85.4)			.89^a^	
	Partnership, n (%)			425 (69.1)			379 (72.1)			46 (51.7)			<.001^a^	
	Language (English), n (%)			602 (97.9)			517 (98.3)			85 (95.5)			.10^c^	
**Population-level characteristics**														
	Median income, mean (SD)			$96,908 ($20,081)			$96,889 ($20,290)			$97,020 ($18,868)			.96^b^	
	Education (proportion>High School), mean (SD)			0.6 (0.1)			0.6 (0.1)			0.6 (0.1)			.58^b^	
**Comorbidities**														
	Depression or anxiety, n (%)			109 (17.7)			93 (17.7)			16 (18.0)			.95^a^	
	Chronic pain, n (%)			39 (6.3)			37 (7.0)			2 (2.3)			.10^c^	
	**Charlson comorbidity index categories, n (%)**													<.001^a^
		0	285 (46.3)			256 (48.7)			29 (32.6)					
		1	159 (25.9)			140 (26.6)			19 (21.4)					
		2	101 (16.4)			78 (14.8)			23 (25.8)					
		>3	70 (11.4)			52 (9.9)			18 (20.2)					

^a^Analyzed using chi-square test.

^b^Analyzed using student *t* test.

^c^Analyzed using Fisher exact test.

### Generalizability

**Figure 2:**
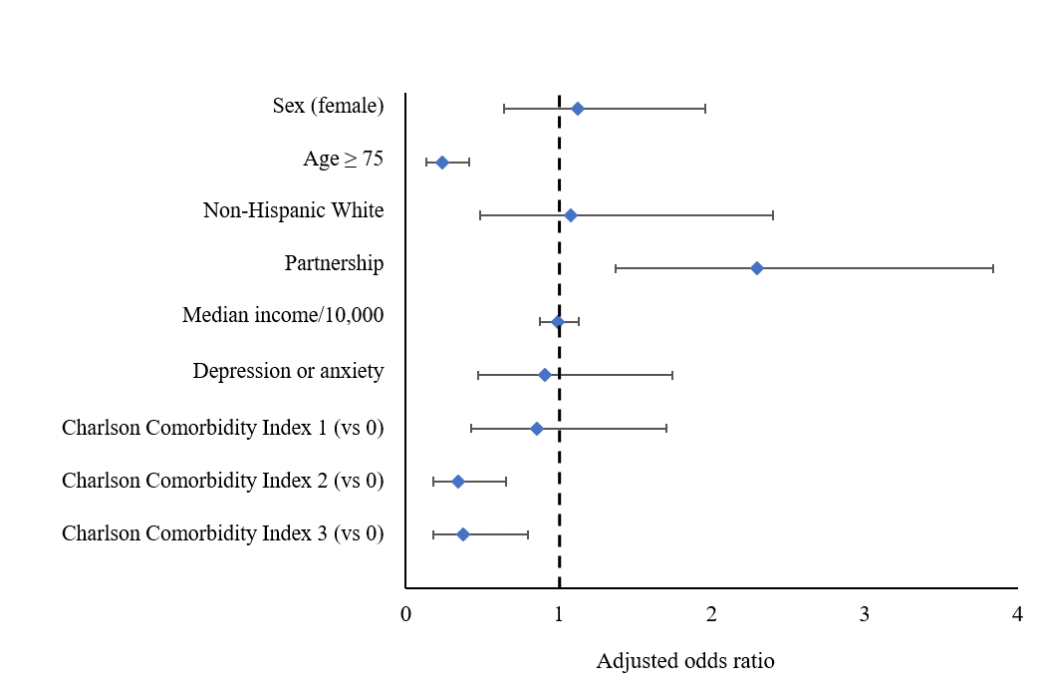
Forest plot display of the adjusted odds ratios and 95% CIs for characteristics of opting in to the follow-up program. Results from the multivariable logistic regression for characteristics of inclusion were displayed (odds ratio and 95% CI) as a forest plot.

In the adjusted analyses, those ≥75 years old were much less likely to participate compared with those aged <75 years (odds ratio [OR] 0.2, 95% CI 0.1-0.4; *P*<.001; Figure 2, Table 2). Those with a higher CCI of either 2 or >3 had lower odds of participating compared with those with a CCI score of 0, and those in a current partnership or marriage had more than twice the odds of participating compared with those who were not (OR 2.2, 95% CI 1.3-3.6; *P*=.002).

**Table 2:** Adjusted odds of opting into the follow-up program.

Characteristics of response			OR^a^ (95% CI)		*P* value
Sex (female)			1.1 (0.7-1.9)		.71
Age (≥75 years)			0.2 (0.14-0.4)		<.001
Race/ethnicity (non-Hispanic white)			1.2 (0.6-2.6)		.61
Partnership			2.2 (1.34-3.6)		.002
Median income (per US $10,000)			1.0 (0.9-1.1)		.92
Depression or anxiety			1.0 (0.5-1.8)		.92
**Charlson comorbidity categories**					
	0	Reference		Reference	
	1	0.9 (0.5-1.8)		.76	
	2	0.4 (0.2-0.7)		.003	
	>3	0.4 (0.2-0.8)		.008	

^a^OR: odds ratio.

### Characteristics of Response

**Figure 3:**
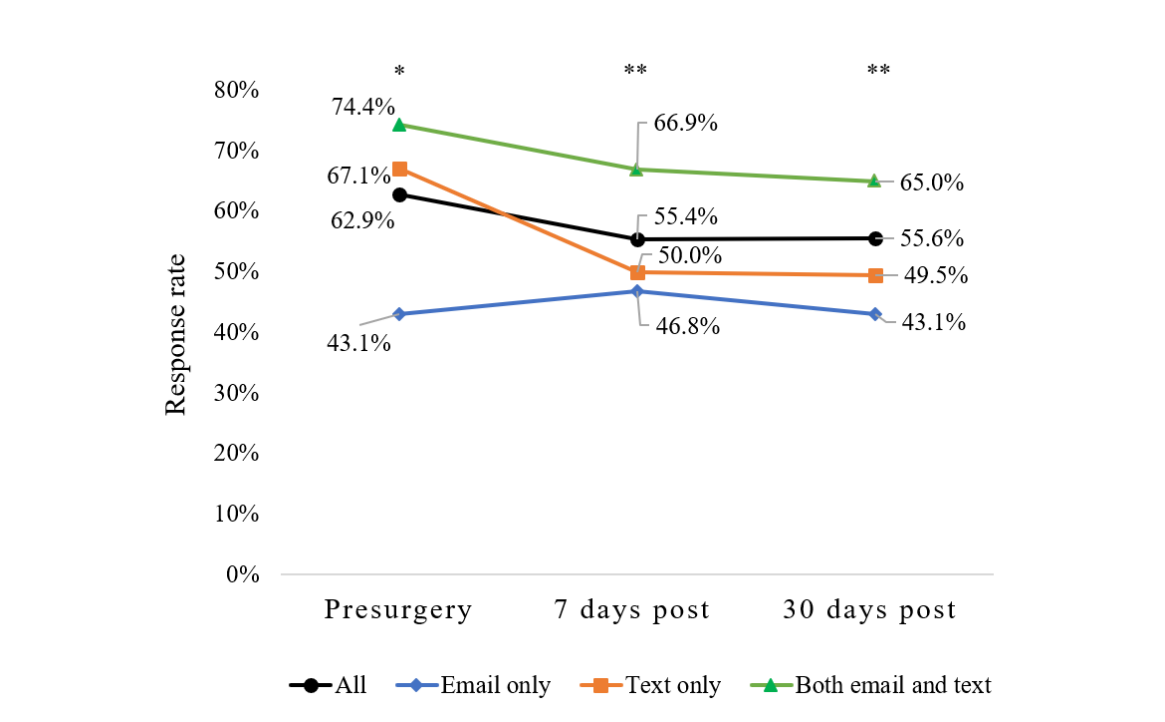
Response rates by method of contact. There were significant differences in response rates according to the method of contact at all 3 time points. The concurrent mixed-mode approach resulted in significantly higher response rates over time compared with single-mode approaches alone. *Chi-square *P* value for the 3-group comparison <.001. **Chi-square *P* value for the 3-group comparison=.001.

Of the 463 patients included in the response analyses, 291 (62.9%) responded to the preoperative survey, 256 (55.4%) responded to the 7-day postoperative survey, and 257 (55.6%) responded to the 30-day postoperative survey. Notably, response rates significantly differed by method of contact at each time point, with the CMM approach having the highest response rates over time compared with the single-mode approaches (Figure 3). This pattern of response by method of contact was mostly consistent within several exploratory subgroup analyses (eg, within subgroups of partnership and race), although differences did not reach statistical significance (results not shown). Rates of response were similar by method of contact for the older age group at the pre and 7-day time points, but CMM was superior at the 30-day time point (results not shown).

**Figure 4:**
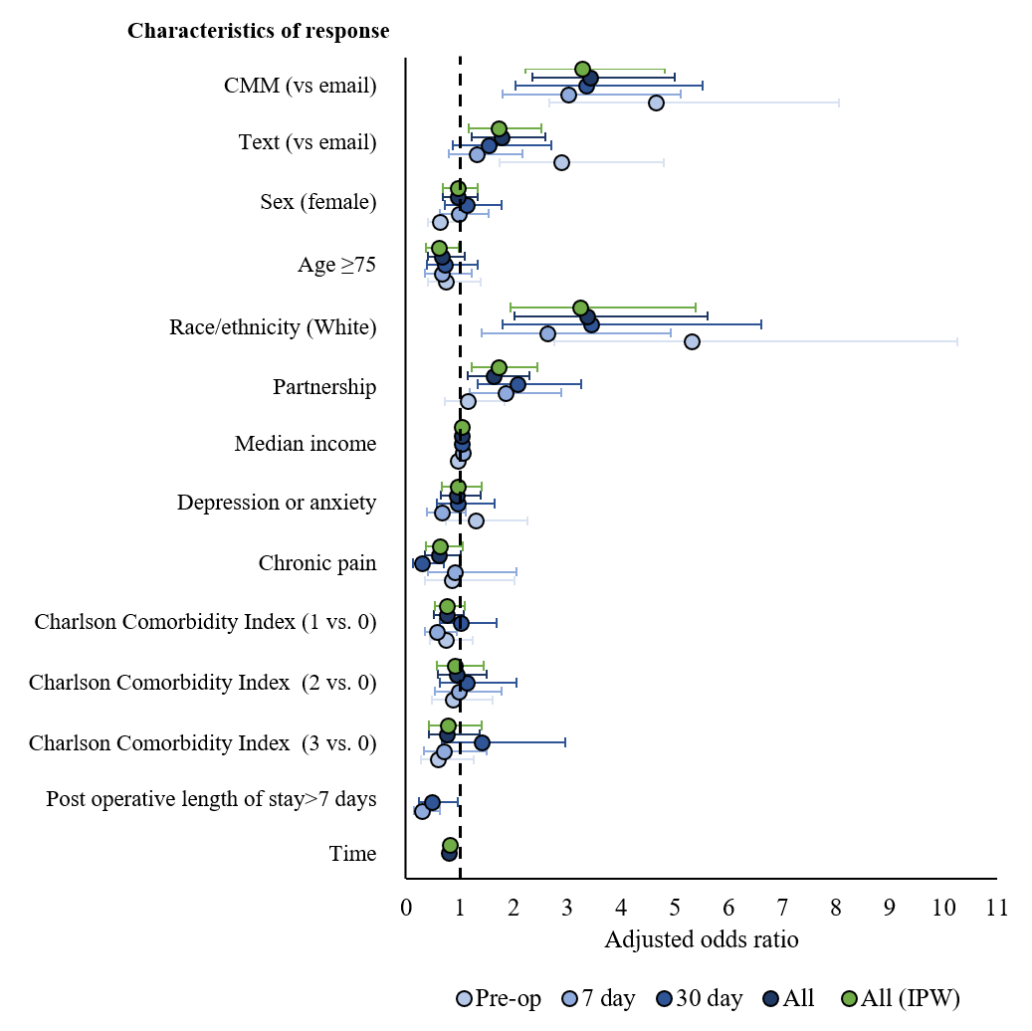
Forest plot display of the adjusted odds ratios (ORs) and 95% CI for characteristics of response (individual time point and combined models). Forest plot results from all 5 adjusted models are shown. The dashed line indicates an OR of 1. Concurrent mixed modes and race were consistently significant positive predictors of response in all the models. Partnership was also a significant positive predictor of response in most models. Age was a significant negative predictor in the inverse probability weighted model. Postoperative length of stay >7 days was a significant negative predictor in the 7- and 30-day models. Time, which was analyzed in the repeated-measures models, also negatively predicted the response. CMM: concurrent mixed modes; IPW: inverse probability weighted.

Multivariable logistic regression results for possible response characteristics are presented for the 3 time points in Table 3, and for all models in Figure 4. In the adjusted models, the method of contact, race, and partnership were the most consistent determinants of response at each time point. After adjusting for all characteristics in the generalized estimating equations analysis (Figure 4; Table 4), using CMM or SMS alone resulted in significantly higher odds of response compared with the email group (CMM OR 3.4, *P*<.001; SMS OR 1.8, *P*=.003). In contrast, CMM also demonstrated significantly higher adjusted odds of response compared with SMS alone (CMM OR 1.9, *P*<.001). The additional significant predictors were race, partnership, and time.

Inverse probability weights were calculated from the generalizability-adjusted logistic regression model and applied to the nonresponse models in a sensitivity analysis (Figure 4*).* Most of the effects and significance levels for the characteristics in each time period model remained the same, indicating that the coverage error had little effect on the response analyses. Decomposition analyses revealed no significant differences in the composition (ie, demographic or clinical characteristics) of the CMM, email alone, and SMS alone groups. All differences in the effects on response were attributed solely to the method of contact.

**Table 3:** Multivariable logistic regression model results for each time point.

Characteristics		Preoperative			7-day postoperative			30-day postoperative	
		OR^a^ (95% CI)	*P* value	OR (95% CI)		*P* value	OR (95% CI)		*P* value
**Method of contact**									
	Email	Reference	N/A^b^	Reference		N/A	Reference		N/A
	Text	2.9 (2.7-8.1)	<.001	1.3 (0.8-2.2)		.29	1.5 (0.9-2.7)		.14
	Both	4.6 (1.7-4.8)	<.001	3.0 (1.8-5.1)		<.001	3.4 (2.0-5.5)		<.001
Sex (female)		0.6 (0.4-1.0)	.06	0.9 (0.6-1.5)		.90	1.1 (0.7-1.8)		.60
Age (≥75 years)		0.7 (0.4-1.4)	.35	0.7 (0.4-1.2)		.18	0.7 (0.4-1.3)		.29
Race ethnicity (White)		5.3 (2.8-10.3)	<.001	2.6 (1.4-4.9)		.003	3.4 (1.8-6.6)		<.001
Partnership		1.2 (0.7-1.8)	.56	1.9 (1.2-2.9)		.006	2.1 (1.3-3.3)		.002
Median income^c^		1.0 (0.9-1.1)	.38	1.1 (0.9-1.2)		.31	1.0 (0.9-1.1)		.62
Depression or anxiety		1.3 (0.7-2.3)	.39	0.7 (0.4-1.1)		.11	0.9 (0.6-1.6)		.88
Chronic pain		0.8 (0.4-2.0)	.70	0.9 (0.4-2.1)		.81	0.3 (0.1-0.7)		.01
**Charlson comorbidity index**									
	0	Reference	N/A	Reference		N/A	Reference		N/A
	1	0.7 (0.5-1.2)	.26	0.6 (0.4-0.9)		.03	1.0 (0.6-1.7)		.93
	2	0.9 (0.5-1.6)	.66	0.9 (0.5-1.8)		.93	1.1 (0.6-2.0)		.69
	>3	0.6 (0.3-1.3)	.17	0.7 (0.3-1.5)		.36	1.4 (0.7-3.0)		.38
In-hospital >7 day		N/A	N/A	0.3 (0.1-0.6)		.003	0.5 (0.2-1.0)		.04

^a^OR: odds ratio.

^b^N/A: not applicable.

^c^Median income has been divided by 10,000 for ease of interpretation.

**Table 4:** Full model generalized estimating equations analysis results for predictors of response.

Characteristics			OR^a^ (95% CI)		*P* value
**Method of contact**					
	Email	Reference		N/A^b^	
	Text	1.8 (1.2-2.6)		.003	
	Both	3.4 (2.3-5.0)		<.001	
Sex (female)			1.0 (0.7-1.3)		.78
Age ≥75 years			0.7 (0.4-1.1)		.11
Race ethnicity (White)			3.4 (2.0-5.6)		<.001
Partnership			1.6 (1.2-2.3)		.01
Median income^c^			1.0 (0.9-1.1)		.47
Depression or anxiety			0.9 (0.6-1.4)		.75
Chronic pain			0.6 (0.4-1.0)		.06
**Charlson comorbidity index**					
	0	Reference		N/A	
	1	0.8 (0.5-1.1)		.11	
	2	0.9 (0.6-1.5)		.78	
	>3	0.8 (0.4-1.4)		.36	
Time			0.8 (0.7-0.9)		<.001

^a^OR: odds ratio.

^b^N/A: not applicable.

^c^Median income has been divided by 10,000 for ease of interpretation.

### Early, Delayed, and Late Responders

There was no evidence of nonresponse bias in the QOR-9 scores (Table 5). 11 surveys were incomplete in the preoperative period and 8 were incomplete in the postoperative period. The surveys were removed from the analysis. Of the remaining 350 patients who responded at the preoperative time point, 212 (60.6%) responded early, 68 (19.4%) responded after the reminder, and 70 (20%) responded via the telephone call. At the 7-day postoperative time point, 301 participants responded with complete surveys: 171 (56.8%) after the first survey, 79 (26.2%) after the reminder, and 51 (16.9%) via the telephone call. Preoperative and 7-day QOR-9 scores were not significantly different between the early, delayed, and late responders (Table 5). Further sensitivity analyses within subgroups of method of contact, partnership, age, and race demonstrated similar findings (results not shown).

**Table 5:** Detecting nonresponse bias in outcomes—Quality of Recovery-9 scores for early, delayed, and late responders.

Responder groups			Value, n (%)		Value, median (IQR)		Kruskal–Wallis *P* value	
**Preoperative**								.73
	All	350 (100)		16 (14-17)				
	Early responders	212 (60.6)		16 (14-17)				
	Delayed responders	68 (19.4)		15 (14-17)				
	Late responders	70 (20)		16 (14-17)				
**7-day postoperative**								.29
	All	301 (100)		15 (13-17)				
	Early responders	171 (56.8)		15 (13-17)				
	Delayed responders	79 (26.2)		15 (13-16)				
	Late responders	51 (16.9)		15 (14-17)				

## Discussion

### Principal Findings

In this one-center enhanced recovery after surgery population, a web-based and mobile communications program appeared to be a feasible approach for collecting longitudinal recovery surveys. Most eligible patients (526/615, 85.5%) opted for the web-based follow-up program, and over half of these patients responded to the survey at each time point. Using CMM to deliver the surveys was an effective strategy to increase response rates compared with email or SMS alone.

However, there were some significant predictors of the inclusion. Those who had a higher number of comorbidities (CCI >1), individuals who were not in a partnership, and individuals over the age of 75 were less likely to opt for the follow-up program. Age was not a significant predictor of response in the adjusted models but became significant after the additional inverse probability weights were applied from the opt-in model. CCI was not a significant predictor of response in any of the adjusted models, including the inverse probability weighted model, whereas partnership was a significant positive predictor in all response models.

Overall response rates declined from the preoperative time point (291/463, 67.1%) to the postoperative time points (256/462, 55.4% and 257/462, 56.6%), but can still be considered relatively high compared with the average response rates for the Hospital Consumer Assessment of Health Care Providers and Systems survey. The Hospital Consumer Assessment of Health Care Providers and Systems survey is a widely used hospital satisfaction survey that uses a combination of mail, telephone, and email to obtain responses from recently discharged patients after surgery and had an average response rate of 23% in New York State in 2018 [26].

The odds of response were significantly higher among those in whom a CMM approach was used to deliver the recovery surveys compared with email alone and SMS alone. These results were consistent over time, even after adjusting for all other risk factors that traditionally predict survey responses. The pattern of CMM achieving the highest response rate over time also remained relatively consistent in different subgroup analyses (eg, within each category of age, race, and partnership). This suggests that using CMM is effective in increasing response rates, even within harder to reach groups. Decomposition analyses confirmed that there were no underlying differences in the observable demographic or clinical characteristics within the 3 method of contact groups that contributed to the differences in response rates, thereby lending credibility to the findings of the response analyses.

There are several possible explanations for why the CMM approach achieved the highest response rates. Giving users a choice to respond with their preferred mode is likely to increase their response [27]. Moreover, receiving the survey prompt to multiple points of contact may have led to an increase in trust. One large survey study in New Zealand explored specific barriers to responses to web-based surveys [28]. Although this survey was conducted in 2009, one of the barriers identified was trust in relation to spam email or survey requests. Participants associated most survey requests with spam and reported routinely ignoring emails that appeared to be unsolicited at the expense of accidentally ignoring genuine requests. Overall, trust was a minor barrier identified in comparison to issues related to time and effort. Nevertheless, email-related spam is still a major issue, with over half of the current emails estimated to be spam [29]. We do not know the frequency at which our email requests were accidentally and automatically funneled into participants’ spam folders, but we reduced this likelihood by using a university email domain. Moreover, by sending the recovery survey to multiple points of contact, we may have increased patients’ trust in the legitimacy of the request.

Nonresponse was not associated with lower education or income in the adjusted models. These results are similar to a texting study that assessed pain at 2 weeks postoperatively [11], but contrary to a previous study that sent web-based PRO surveys through email [15]. The latter study consisted of women only but was more nationally representative of the study population. The overall geographic area examined in our study was fairly wealthy and educated, which could explain this difference. The median household income in the United States was approximately US $63,000, whereas the median household income in our study was approximately US $97,000.

Race was associated with nonresponse, which is consistent with previous studies [15]. Specifically, those who were Hispanic/non-White had consistently lower odds of responding to the surveys. To understand what effect this might have on interpreting the QOR-9 scores from responders, a separate subgroup analysis was performed to evaluate the risk of nonresponse bias by comparing QOR-9 scores in early, delayed, and late responders who were Hispanic/non-White. Within this subgroup analysis, there were no differences in QOR-9 scores between early, delayed, and late responders at either the preoperative time point or the 7-day time point. However, the sample sizes in these subgroups were small. Nonetheless, these null findings provide modest evidence that even though response rates were lower in this subgroup, those who responded may be representative of the full subgroup.

In fact, no differences were found in the overall and subgroup analyses comparing early, delayed, and late responder recovery scores. Under the continuum of resistance model, nonresponders are most similar to those who require more reminders to complete the survey [30]. Since our recovery scores do not differ between early, delayed, and late respondents, it is possible that nonresponders have similar scores to those included in the sample.

This study has several limitations. First, even though we can describe the patients who did not want to participate (ie, opt-in), we do not know the exact reasons for nonparticipation. It is possible that patients had limited access to technology or that patients simply did not want to participate. We also did not randomize the patients who participated in the 3 method of contact groups. However, decomposition analyses helped alleviate the concern that these groups were somewhat different in composition of characteristics. Indeed, the composition of the groups was similar, and there did not appear to be trends in characteristics of patients over time (eg, older patients were no more or less likely to have surgery in June compared with November).

Approximately 18% (82/463) of patients received the surveys through a different method of contact other than the one preassigned. This was for various reasons: of the 463 participants, 24 (5.2%) participants in either phase 1 or 3 did not provide an email, and were therefore sent surveys by text alone; 31 (6.7%) participants either in phase 2 or 3 did not own a smartphone, and were therefore sent surveys through email alone; 2 (0.4%) participants in phase 2 did not list a phone number and were sent surveys through email alone; and 25 (5.4%) in phase 2 or 3 either provided contact information that was illegible, or a separate system error occurred in which participants received emails alone. A separate system error was also responsible for shifting several participants into the email and text group at 30 days instead of receiving text or email alone, but this was accounted for in the analyses. Nevertheless, this demonstrates that a system that is preprogrammed to send surveys concurrently through both email and text could prevent the exclusion of individuals who either did not have a smartphone or did not provide an email address by casting a broader net.

Non-White individuals accounted for only 13.3% (82/615) of our sample, most of whom were Hispanic or African Americans. There were too few participants in either the independent race/ethnicity group to perform significance testing separately, but independent examinations did not reveal any inconsistencies between the individual groups and the combined group. The lack of any detected nonresponse bias in recovery scores lends some credence to the representativeness of the sample, but it would still be important to increase response rates among non-White participants and increase the sample size. Furthermore, using late responders as a proxy for nonresponders provides some evidence that nonresponders were similar to responders, but actual scores of nonresponders could not be assessed. In addition, multiple types of surgical procedures were included within this study, which made it impossible to include the procedure as a covariate. Length of stay was used as a proxy measure for the severity of the surgical procedure and (or) in-hospital complications, but specific surgical area studies could be explored to verify our study findings. Area-level metrics were used as proxies for individual income and education metrics and were much higher than the national averages. These may not truly reflect income and education at the individual level and may also have limited our understanding of how these characteristics affect both inclusion and response.

This study had several strengths. The email or text capture system was applied to the QA program. Therefore, we were able to identify issues of practicality that clinicians might face when attempting to capture postdischarge recovery data in a broad patient population. This provided an opportunity for an in-depth analysis of the generalizability and response rates when using email or text-based programs in surgical patients. At the time, Twilio SMS messages cost US $0.0075 per message. Therefore, we also demonstrated a resource-friendly method that can obtain high response rates in the early postoperative period. This is important as many complications that occur early after discharge result in readmission [31]. Although we did not actively monitor patient responses, this type of system could in fact be reprogrammed to do so. Although patient surveys contained no identifiable information, they were linked back to a secured database with patient information. Specified algorithms could be developed to send alerts to medical staff regarding patients who appear to be developing a complication or have other unmet medical needs. This would provide an opportunity to intervene before readmission.

### Conclusions

A web-based and mobile communication program appears to be a feasible approach for collecting longitudinal perioperative recovery surveys in the ERAS population. Additional efforts may be required to increase participation within non-White individuals, older individuals, and those who are not in a partnership. However, using a CMM approach is an effective strategy to reduce nonresponse, even in difficult-to-reach subgroups. Finally, a web-based system such as the one described could be a cost-effective approach to improve communication between the patient and clinician during a period in which the communication is lacking.

## Abbreviations

CCI: Charlson Comorbidity Index
CMM: concurrent mixed modes
ERAS: enhanced recovery after surgery
IPW: inverse probability weighted
OR: odds ratio
PRO: patient-reported outcomes
QA: quality assurance
QOR-9: Quality of Recovery-9
